# Associations between state intimate partner violence-related firearm policies and injuries among women and men who experience intimate partner violence

**DOI:** 10.1186/s40621-021-00297-y

**Published:** 2021-02-22

**Authors:** Tiara C. Willie, Trace Kershaw, Rachel Perler, Amy Caplon, Marina Katague, Tami P. Sullivan

**Affiliations:** 1 Department of Mental Health, Johns Hopkins Bloomberg School of Public Health, Baltimore, MD USA; 2 Department of Social and Behavioral Sciences, Yale School of Public Health, New Haven, CT USA; 3 National Institutes of Health, National Cancer Institute, Bethesda, MD USA; 4 Department of Psychiatry, Yale University School of Medicine, New Haven, CT USA

**Keywords:** Intimate partner violence, Firearms, Injuries

## Abstract

**Background:**

Comprehensive state firearm policies related to intimate partner violence (IPV) may have a significant public health impact on non-lethal IPV-related injuries. Research indicates that more restrictive firearm policies may reduce risk for intimate partner homicide, however it is unclear whether firearm policies prevent or reduce the risk of non-lethal IPV-related injuries. This study sought to examine associations between state-level policies and injuries among U.S. IPV survivors.

**Methods:**

Individual-level data were drawn from the National Intimate Partner and Sexual Violence Survey, a nationally-representative study of noninstitutionalized adults. State-level data were drawn from a firearm policy compendium. Multivariable regressions were used to test associations of individual policies with non-fatal IPV-related injuries (*N* = 5493). Regression models were also conducted to explore differences in the policy-injury associations among women and men survivors.

**Results:**

Three categories of policies were associated with IPV-related injuries. The odds of injuries was lower for IPV survivors living in states that prohibited firearm possession and require firearm relinquishment among persons convicted of IPV-related misdemeanors (aOR [95% CI] = .76 [.59, .97]); prohibited firearm possession and require firearm relinquishment among persons subject to IPV-related restraining orders (aOR [95% CI] = .81 [.66, .98]); and prohibited firearm possession among convicted of stalking (aOR [95% CI] = .82 [.68, .98]) than IPV survivors living in states without these policies. There was a significant difference between women and men survivors in the association between IPV-related misdemeanors policy and injuries (B [SE] = .60 [.29]), such that the association was stronger for men survivors (aOR [95% CI] = .10 [.06, .17]) than women survivors (aOR [95% CI] = .60 [.48, .76]).

**Conclusions:**

Restrictive state firearm policies regarding IPV may provide unique opportunities to protect IPV survivors from injuries.

## Introduction

Intimate partner violence (IPV) is a critical public health problem that can have significant effects on population health (Devries et al. 2013; Garcia-Moreno et al. 2006). In the United States (U.S.), more than one in three women (36.4%) and men (33.6%) experience some form of IPV in their lifetime (Smith et al. 2018). An extensive body of research illustrates the adverse physical, mental, sexual and reproductive health (Miller et al. 2010; Decker et al. 2013) outcomes associated with experiencing IPV. Nonfatal injuries resulting from victimization are prevalent concerns for women and men who experience IPV. For example, it is estimated that 41% of women and 14% of men who experience IPV report some form of physical injury (Centers for Disease Control Prevention 2010). These nonfatal injuries can range from minor abrasions such as scratches and welts to more serious injuries such as broken bones and head injuries (National Center for Injury Prevention and Control 2003; Sheridan and Nash, 2007). Previous research indicates that nonfatal injuries can be caused by multiple sources such as blunt objects (e.g., closed fist), weapons (e.g., firearms) and household objects (e.g., kitchen appliance) (Sheridan and Nash, 2007). Nonfatal injuries can have a long-lasting impact on survivors’ wellbeing such as long-term disability (National Center for Injury Prevention and Control 2003; Black 2011); and it can also result in significant societal costs associated with medical services and lost productivity (National Center for Injury Prevention and Control 2003). Identifying and implementing evidence-informed strategies that reduce nonfatal injuries related to IPV victimization is an important, understudied topic.

IPV-related firearm policies may be critical evidence-informed strategies to reduce nonfatal injuries among IPV survivors in the U.S. There are several advances in federal legislation designed to remove firearms and prevent potentially dangerous intimate partners from using firearms to cause injury. For example, under the Violence Against Women Act (1994), individuals are prohibited from firearm possession if they are subject to an IPV-related restraining order (1994). Federal policies also prohibit firearm possession among persons convicted of felonies; and in the 1996 amendment to the Gun Control Act, the prohibition was extended to misdemeanors (Díez et al. 2017). While these policies prevent certain intimate partners from possessing firearms, previous research has underscored the importance of the “relinquishment gap”(Díez et al. 2017; Giffords Law Center to Prevent Gun Violence, 2020a, b). Identified by the Law Center to Prevent Gun Violence, the “relinquishment gap” is a loophole in the federal law that does not explicitly require certain partners from relinquishing or surrendering their firearms (Díez et al. 2017; Giffords Law Center to Prevent Gun Violence, 2020a, b). Federal law sets the standard for state policies, but states may enact more stringent laws and some states have gone beyond federal law to require removal firearms from potentially dangerous intimate partners. For example, some states have firearm possession prohibition policies for those subject to stalking misdemeanors and from dating partners subject to IPV-related misdemeanors and protective orders (Zeoli et al. 2017). IPV-related firearm policies that address stalking are vital as stalking behaviors are common among perpetrators of IPV (Logan et al. 2007). Further, even though federal law only applies to a partner who is a spouse or have a child in common with the victim, dating partners may also engage in violence with a firearm and some states have taken additional steps to cover this group of perpetrators in firearm policies (Zeoli, 2018a, b). Finally, some states require that law enforcement remove firearms from the scene of an IPV incident which could also prevent firearms from being used to inflict injury in the near future. Collectively, the heterogeneity in state IPV-related firearm policies may differentially impact the wellbeing of victims based on their state of residence.

The association between state firearm policies related to IPV and nonfatal injuries has yet to be examined, but a significant body of research has examined its association with intimate partner homicide, the most severe outcome of IPV. Half of intimate partner homicides in the U.S. are committed with firearms (Díez et al. 2017), and 85% of victims are women (McFarlane et al. 1999). Further, several studies found that more restrictive state IPV-related firearm policies are associated with reductions in intimate partner homicide (Díez et al. 2017; Zeoli et al. 2019; Gollub and Gardner, 2018; Sivaraman et al. 2019). For example, Díez et al. (2017) found that states that prohibited firearm possession and also required firearm relinquishment among individuals subject to IPV-related restraining orders had lower rates of intimate partner homicide than states without these policies. Sivaraman et al. (2019) found that more restrictive state firearm policies were associated with lower rates of women intimate partner homicides. Collectively, these studies illustrate that removing firearms from firearm owners involved in relationships where IPV is present may prevent intimate partner homicide because these individuals cannot use their firearms as weapons against their intimate partners. This reasoning may also be relevant for the prevention of nonfatal injuries. In the context of nonfatal injuries, firearms can be used to exert power and control dynamics within a relationship (Sullivan and Weiss, 2017). An intimate partner’s access to firearms has been associated with more severe IPV (McFarlane et al. 1998; Zeoli et al. 2016) and firearm-related threats (Rothman et al. 2005). Therefore, removing an intimate partner’s access to firearms may prevent these partners from using their firearm as a weapon for nonfatal injury (e.g., using it to inflict blunt force-related injuries).

State IPV-related firearm polices may directly reduce nonfatal injuries by removing firearm access, but these policies may also *indirectly* impact nonfatal injuries. According to the social ecological model (National Center for Injury Prevention and Control 2015), a theory widely used in violence prevention research, state IPV-related policies are structural factors that create and maintain climates in which IPV is legitimized or prohibited, and these climates may, in turn, affect the wellbeing of individuals who experience IPV. Building from this theoretical model, there are some potential ways in which IPV-related firearm policies could disrupt the mechanism of violence-related injury (i.e., the exchange of physical forces that result in injury) (Sheridan and Nash, 2007). First, firearms are used by intimate partners in nonfatal violent incidents (Planty and Truman, 2013), and policies that restrict firearm possession and require firearm relinquishment may reduce the chances of a firearm being used as a weapon against victims of IPV. Second, IPV-related firearm policies may also, *indirectly*, reduce the chances of a perpetrator using a nonfirearm weapon or object (e.g., knives) to cause a nonfatal injury. Firearms can be used as a way to exert coercive control in a relationship (Sorenson and Schut, 2018; Sorenson 2017) and firearm access is associated with more assaults that did not involve a gun (Zeoli et al. 2019; Folkes et al. 2013). Restricting and removing firearm access may reduce a perpetrator’s ability to exert coercive control, and thus result in fewer injuries. This rationale is consistent with a previous study that found firearm use to be associated with fewer visible physical injuries among victims but more threats and fear among victims (Sorenson 2017). Further, previous research also suggests that firearm access is associated with an increased likelihood of using other objects and weapons to commit an assault (Zeoli et al. 2016; Folkes et al. 2013). IPV perpetrators with firearm access may have access to other weapons, however it is possible that the legal consequences in regard to restricting and removing firearms from IPV perpetrators (e.g., being arrested, being “served”, surrendering firearms to law enforcement or gun dealer) may reduce or deter a perpetrator’s motivation to inflict injury to an intimate partner. This rationale is consistent with an emerging body of research indicating that greater awareness and involvement with the legal consequences related to IPV were associated with lower incidents of subsequent IPV acts (Song et al. 2017; Lyons et al. 2019).

Although it is unclear whether state IPV-related firearms are associated with better health outcomes among women and men who experience IPV, prior research on state IPV-related policies suggest that these types of policies may improve health outcomes by functioning as “health-related policies.” The CDC describes “health-related policies” as the “formal or informal written statements that are designed to protect or promote health”(Centers for Disease Control and Prevention 2015). Previous research suggests that state IPV-related policies shape HIV transmission among women. Specifically, a study found that the positive association between IPV prevalence and HIV diagnoses was significantly attenuated in states with more IPV-related healthcare policies compared to states with fewer policies (Willie et al. 2018). Notably, this study suggested that state policies were associated with women’s HIV vulnerability such that trauma-informed healthcare environments create safe spaces to identify women experiencing IPV and better address their health concerns. Building from prior work on state IPV-related policies, IPV survivors residing in states with more restrictive firearm possession prohibition policies may have better health outcomes, such as fewer nonfatal injuries.

The current study aimed to examine the association between IPV-related firearm policies implemented at the state level and nonfatal injuries among individuals who experience IPV. We hypothesized that individuals who experience IPV living in a state with more restrictive firearm possession prohibition policies would have lower odds of nonfatal injuries than individuals who experience IPV living in states with less restrictive firearm possession prohibition policies. In this study, more restrictive firearm possession prohibition policies refers to a comparison between states that: (1) prohibit firearm possession by persons subject to IPV-related restraining orders and also (2) require firearm relinquishment compared to states with no prohibition, which are considered less restrictive. Given the dearth of research on the differential associations of individual policies and gender, these relationships were explored, and no specific hypotheses were advanced.

## Methods

### Study sample

Individual-level data were drawn from the National Intimate Partner and Sexual Violence Survey (NISVS), a cross-sectional, nationally representative epidemiologic survey of the non-institutionalized English and Spanish-speaking U.S. population aged 18+ (Black et al. 2011). The survey was conducted in 50 states and the D.C. and administered from January 2010 to December 2010. NISVS is a random digit dial (RDD) telephone survey with dual-frame sampling that assessed multiple forms of interpersonal violence including IPV, sexual violence, and stalking among women and men. The NISVS was constructed to provide both state-level and national-level estimates for multiple forms of interpersonal violence. A total of 18,049 interviews were conducted using landline telephones (45.2%) and respondents’ cell phones (54.8%). The study protocol was approved by the Office of Management and Budget (OMB# 0920–0822) and the Institutional Review Board of the Research Triangle Institute, International. Further information on the study design, training, data collection, and study implementation are published elsewhere (Black et al. 2011).

The current study was exempted by [Institution masked for peer review] IRB because the focus was secondary data analysis of de-identified data. The current analyses were restricted to women and men who experienced lifetime physical, sexual, and/or psychological IPV without missing data for nonfatal injury (*N* = 5493). This data was analyzed in 2020.

### Measures

#### State-level data

##### State IPV firearm policies

We coded the presence and absence of six categories of state-level firearm statutes related to IPV extracted from the Everytown for Gun Safety database (Everytown for Gun Safety 2014), a domestic violence and firearms compendium from the Law Center to Prevent Gun Violence (Giffords Law Center to Prevent Gun Violence, 2020a, b), and the Westlaw and Lexis Nexis databases, which have been consistently used in previous research (Díez et al. 2017; Zeoli et al. 2019; Gollub and Gardner, 2018). The six categories of statutes are: 1) Prohibition of firearm possession by persons convicted of IPV-related misdemeanors, with and without firearm relinquishment; 2) Prohibition of firearm possession by persons subject to IPV-related restraining orders, with and without firearm relinquishment; 3) Prohibition of firearm possession by persons convicted of stalking misdemeanors; 4) Removal of firearms from the scene of an IPV incident; 5) Prohibition of firearm possession by dating partners convicted of IPV-related misdemeanors; and 6) Prohibition of firearm possession by dating partners convicted of IPV-related protective orders. For each policy, states were coded as a 1 (presence) or 0 (absence). The coding was based on 2010 policies and legislations given the timeframe of the data collection of the NISVS. The lead author coded the state-level statutes initially, and three co-authors double-coded these variables to ensure that the statutes were coded correctly. Due to the firearm relinquishment requirement, three of the six categories of statutes were coded as three-level categorical variables. For example, for the Prohibition of firearm possession by persons convicted of IPV-related misdemeanors variable, states were coded as: 0 = prohibition statute was absent, 1 = prohibition statue was present without firearm relinquishment requirement, and 2 = prohibition statute was present with firearm relinquishment requirement. The remaining three categories of statutes were coded as binary variables such that: 0 = statute was absent and 1 = statute was present.

##### State covariates

We also controlled for the prevalence of firearm ownership and violent crimes. State firearm ownership was measured as the percentage of suicides committed with firearms for each state (Siegel et al. 2013; Azrael et al. 2004). Suicide data was collected from the Centers for Disease Control and Prevention’s Web-Based Injury Statistics Query and Reporting Systems database (Centers for Disease Control Prevention 2015). State violent crimes was measured as the percentage of violent crimes reported to law enforcement (i.e., murder, rape, robbery and aggravated assault) for each state. Violent crime data was collected from the Uniform Crime Reporting Statistics database. All state-level variables were linked to individual-level data in the NISVS dataset.

#### Individual-level data

##### Nonfatal injuries

NISVS assessed participant report of injuries as a result of an experience of IPV (e.g., “Were you ever injured when this/any of these things happened with any of these people?”). Participants were able to respond as Yes, No, and I don’t know. For the current analysis, this variable was dichotomized as Yes (1) vs. No (0). Responses of “I don’t know” were removed from the analytic sample.

##### Socio-demographics

Participants were asked to self-report socio-demographics: gender (women, men); age (< 10, 11–17, 18–24, 25–34, 35–44, 45–54, and 55+); education (no schooling, 1-8th grade, some high school, high school graduate, technical or vocational school, some college, college graduate, postgraduate), ethnicity (Hispanic, not Hispanic); and race and ethnicity (Black, White, Hispanic, Asian, Native Hawaiian or Other Pacific Islander, American Indian or Alaska Native, and Other).

#### Analysis

Frequencies of socio-demographics were calculated for women and men who experience IPV. Weighted logistic regression models were conducted to examine associations between each specific IPV firearm policies and nonfatal injuries independently. In particular, a separate model was conducted for each policy. The weighted analyses took into account the dual sampling frames and stratified sampling. Moderation was also assessed by adding product terms between policy variables and gender variable (women vs. men). All models included age, race and ethnicity, and education. All analyses were conducted in 2020 using SAS to account for the complex sample design.

## Results

Table 1 displays the characteristics of the analytic sample of NISVS women and men participants who reported IPV (*N* = 5493). Half of the participants were aged 45 or older, had completed high school, and identified as Non-Hispanic white.

**Table 1:** Demographic Characteristics of NISVS Participants Who Experienced IPV, United States, 2010

Characteristics	Women (*n* = 3501)	Men (*n* = 1982)
**Age**		
18–24	341 (9.7)	262 (13.2)
25–44	1203 (34.4)	767 (38.6)
45 +	1957 (55.8)	953 (48.1)
**Race and Ethnicity**		
Non-Hispanic Black	328 (9.4)	199 (10.0)
Hispanic	256 (7.3)	175 (8.8)
Non-Hispanic Another Race	249 (7.1)	151 (7.6)
Non-Hispanic White	2664 (76.1)	1451 (73.2)
**Education**		
< High School	256 (7.3)	180 (9.6)
High School Graduate	802 (22.9)	571 (28.7)
> High School	2447 (69.9)	1236 (61.7)

*Note.* Number of participants and frequencies are unweighted

Table 2 displays the state-level distribution of the firearm policies in 2010. Among states with at least 1 policy, the three most common policies were prohibition of firearm possession for dating partners subject to IPV-related protective orders (48.0%), prohibition of firearm possession for persons convicted of stalking (48.0%), and prohibition of firearm possession for IPV-related restraining orders and relinquishment of firearms (37.8%).

**Table 2:** IPV-Related Firearm Policies by State in 2010

State	Prohibition of firearm possession by persons subject to IPV-related restraining orders	Prohibition of firearm possession by persons subject to IPV-related restraining orders and required to relinquish firearms	Prohibition of firearm possession by persons convicted of IPV-related misdemeanors	Prohibition of firearm possession by persons convicted of IPV-related misdemeanors and relinquish firearms	Prohibition of firearm possession by dating persons convicted of IPV-related protective orders	Prohibition of firearm possession by dating persons convicted of IPV-related misdemeanors	Prohibition of firearm possession by persons convicted of stalking	Removal of firearms from the scene of an IPV incident
**AK**					**✔**			
**AL**							**✔**	
**AR**							**✔**	
**AZ**					**✔**	**✔**	**✔**	
**CA**		**✔**		**✔**	**✔**		**✔**	**✔**
**CO**							**✔**	
**CT**		**✔**		**✔**	**✔**	**✔**		
**DC**		**✔**		**✔**	**✔**	**✔**		
**DE**	**✔**		**✔**		**✔**		**✔**	
**FL**	**✔**							
**GA**								
**HI**		**✔**		**✔**	**✔**	**✔**		
**IA**		**✔**		**✔**				
**ID**								
**IL**		**✔**	**✔**			**✔**	**✔**	**✔**
**IN**			**✔**				**✔**	
**KS**								
**KY**								
**LA**								
**MA**		**✔**		**✔**	**✔**		**✔**	
**MD**		**✔**	**✔**				**✔**	
**ME**	**✔**							
**MI**								
**MN**				**✔**			**✔**	
**MO**								
**MS**								
**MT**					**✔**			**✔**
**NC**		**✔**			**✔**			
**ND**					**✔**			
**NE**			**✔**			**✔**		**✔**
**NH**		**✔**			**✔**			
**NJ**	**✔**		**✔**		**✔**	**✔**	**✔**	**✔**
**NM**								
**NV**								
**NY**		**✔**	**✔**		**✔**	**✔**	**✔**	
**OH**								**✔**
**OK**								**✔**
**OR**								
**PA**				**✔**	**✔**		**✔**	**✔**
**RI**							**✔**	
**SC**							**✔**	
**SD**			**✔**					
**TN**		**✔**		**✔**				**✔**
**TX**	**✔**		**✔**		**✔**	**✔**	**✔**	
**UT**								**✔**
**VA**								
**VT**								
**WA**		**✔**	**✔**		**✔**	**✔**		
**WI**		**✔**					**✔**	
**WV**	**✔**		**✔**		**✔**	**✔**		**✔**
**WY**								

Table 3 presents the adjusted associations of each individual IPV-related firearm policy with nonfatal injuries. Individuals living in states that prohibited firearm possession and required firearm relinquishment among persons convicted of IPV-related misdemeanors (aOR [95% CI] = .76[.59, .97], *p* = .01), prohibited firearm possession and required firearm relinquishment among persons subject to IPV-related restraining orders (aOR [95% CI] = .81[.66, .98], *p* = .03), and states that prohibited firearm possession among persons convicted of stalking (aOR [95% CI] = .82[.68, .98], *p* = .02) had lower odds of reporting injuries than individuals living in states without these policies.

**Table 3:** Adjusted Associations Between IPV Firearm Policy Climate and Nonfatal Injuries: NISVS Participants Who Experience IPV, United States, 2010

Independent Variables	aOR (95% CI)	*p*-value
**Prohibition of firearm possession by persons convicted of IPV-related misdemeanors**	REF	
No Firearm relinquishment	1.04 (.84, 1.30)	0.09
Firearm relinquishment	.76 (.59, .97)	0.01
**Prohibition of firearm possession by persons subject to IPV-related restraining orders**	REF	
No Firearm relinquishment	1.01 (.76, 1.33)	0.39
Firearm relinquishment	.81 (.66, .98)	0.03
**Prohibition of firearm possession by persons convicted of stalking**	.82 (.68, .98)	0.02
**Removal of firearms from the scene of an IPV incident**	.90 (.74, 1.10)	0.30
**Prohibition of firearm possession by dating persons convicted of IPV-related misdemeanors**	1.08 (.88, 1.34)	0.44
**Prohibition of firearm possession by dating persons convicted of IPV-related protective orders**	.83 (.67, 1.00)	0.05

*aOR* Adjusted odds ratio, *CI* Confidence interval. Adjusted analyses included individual-level characteristics (i.e., age, gender, race and ethnicity, and education) and state-level characteristics (i.e., firearm ownership, violent crime)

We explored gender (women vs. men) as a moderator on the relationships between individual firearm policies and nonfatal injuries (no table shown). There was a significant difference between women and men survivors in the association between IPV-related misdemeanors policy and injuries (B [SE] = .60 [.29], *p* = .04), such that the association was stronger for men survivors (aOR [95% CI] = .10 [.06, .17], *p* < .01) than women survivors (aOR [95% CI] = .60 [.48, .76], *p* < .01). Gender did not significantly moderate the other policy-injury associations.

## Discussion

To our knowledge, the present study is the first study to examine the implications of IPV-related firearm policies and nonfatal injuries among individuals who experience IPV. In general, our findings indicate that IPV-related firearm policies that prohibited possession and required relinquishment of firearms are associated with lower odds of nonfatal injuries. Specifically, individuals living in states with policies that prohibited firearm possession and relinquishment among individuals issued IPV-related misdemeanors and restraining orders had a lower odds of nonfatal injuries than individuals in states without these policies. Also, individuals living in states with policies that prohibited firearm possession among individuals convicted of stalking had a lower odds of nonfatal injuries than individuals living in states without these policies. Our findings suggest that restricting firearm access and possession among individuals subject to IPV-related restraining orders, convicted of IPV-related misdemeanors and stalking may be particularly important to prevent nonfatal injuries among IPV survivors. This evidence underscores the importance of protective policies related to IPV that have the potential to improve survivors’ wellbeing. Compared to downstream approaches that focus on individual treatments, upstream approaches such as restrictive firearm possession prohibition policies may function as population-level strategies to prevent nonfatal injury among survivors of IPV.

In the present study, we expanded previous research by examining the associations of IPV-related firearm policies with nonfatal injuries. Generally, these findings suggest that restricting and removing firearms from perpetrators may reduce nonfatal injuries among survivors. State IPV-related firearm policies can restrict firearm access among those subject to IPV-related restraining orders and convicted of misdemeanors, but these policies may also help high-risk perpetrators recognize the severity of their charge and thus are deterred from inflicting injuries. For intimate partners with firearms who are subject to IPV-related restraining orders and convicted of misdemeanors, the legal consequences related to implementing these firearm policies may deter motivations for inflicting injury and subsequent violence. The current study is unable to test these potential mechanisms, thus it would be useful for future studies to examine these potential mechanisms more explicitly at the individual-level with perpetrators convicted of IPV-related misdemeanors and restraining orders residing in these states with varying degrees of restrictive firearm possession prohibition and relinquishment requirements.

Overall, our results suggest that state policies that prohibited firearm possession and required firearm relinquishment by perpetrators may prevent nonfatal injuries for *both* women and men, however gender significantly moderated one policy-injury association. In particular, the protective association of state policies prohibiting firearm possession and also required firearm relinquishment among persons convicted of IPV-related misdemeanors with nonfatal injuries was stronger for men than women. The characteristics of IPV cases may offer one potential explanation for this significant finding. Across 16 large U.S. counties, 84% of IPV cases involved women victim-men defendant, 12% involved men victim-women defendant, and 4% involved same-gender victim-defendant (Smith and Farole Jr, 2009). Misdemeanors were the most prevalent charge against a defendant, and a weapon was used in nearly a fourth of misdemeanor cases. Although men victim-women defendant cases were uncommon, women defendants were more likely to use a weapon such as a knife or blunt object (Smith and Farole Jr, 2009). Therefore, if firearm possession prohibition policies indirectly deter a perpetrator’s motivation to inflict injury with nonfirearm weapons, then it is possible that this protective association would be more beneficial in men victim-women defendant IPV cases. While this potential explanation is speculative, it is important to note that women defendants are often experiencing victimization in their relationships and their weapon use is often motivated by self-defense (Melton and Belknap, 2003). It would be useful for future research to examine the risk of injury among victims after men and women defendants are charged with IPV-related misdemeanors in states with firearm possession prohibition policies to provide more context into gender-specific mechanisms that may contribute to these associations.

It is worth noting that our findings highlight the importance of firearm policies specific to stalking. Our results indicate that policies prohibit firearm possession in stalking cases were related to lower odds of injuries. Stalking is commonly used by perpetrators of IPV (Logan et al. 2007) and is strongly associated with more severe, and potentially lethal forms of violence (McFarlane et al. 1999; McFarlane et al. 1998). Perpetrators who engage in stalking behaviors may be particularly dangerous to one’s safety and longevity; yet, there is no federal law that prohibits firearm possession for perpetrators who stalk and few states have legislation in place (Zeoli, 2018a, b). The potential implications of this finding are critically important as researchers have called for more attention regarding intimate partner stalking (Logan et al. 2007; McFarlane et al. 1999; McFarlane et al. 1998).

Collectively, our findings support the notion that IPV-related firearm policies are health-related policies, as illustrated by showing that IPV-related firearm policies were associated with better wellbeing in the form of lower odds of nonfatal injuries. Upstream approaches to nonfatal injury prevention through supportive health-related policies have the potential to affect large groups of people simultaneously while fostering a culture of health (Centers for Disease Control and Prevention 2015). Coupled with previous research, our findings suggest that adoption of firearm possession prohibition policies with firearm relinquishment requirements at the state level may improve the health of victims of IPV.

### Limitations

These findings should be interpreted considering the study’s limitations. The NISVS is an ongoing nationally representative survey and although the second wave of data collection is complete, the subsequent wave was not available at the time this manuscript was developed. As a result, our findings are based on cross-sectional data. The cross-sectional data do not allow for causal inferences. Future research should build upon our findings with the additional waves of data collection because longitudinal examinations can examine how policy changes influence health and wellbeing. To date, there is no national surveillance system that tracks nonfatal injuries related to IPV, but NISVS provides a unique opportunity to tackle some important research questions. The nonfatal injury outcome was specific to a participant’s report of IPV only and not time-specific. For example, participants who experienced IPV at multiple points in their lifetime (i.e., past year and past 3 years) were asked the nonfatal injury outcome question and the question was not specific to the past year or past 3 years. Therefore, we were unable to examine the associations between IPV-related firearm policies and nonfatal injuries within the past year. Since these findings are based on a cross-sectional study, it is possible that some state-level confounders (e.g., nonfirearm policies) may have accounted for some of the statistically significant findings. Also, the presence or absence of a policy does not speak to the strength and/or enforcement in the state. It would be useful for future research to measure the implementation of IPV-related policies and how they influence health outcomes among women who experienced IPV. Our analysis does not allow us to assess mechanisms through which IPV-related firearm policies reduce injuries. It would be useful for future research to explore potential mechanisms linking IPV-related firearm policies to injuries. All the study variables were self-reported. Since IPV reporting can be influenced by social desirability bias, the prevalence of IPV may be misclassified.

## Conclusions

The current study adds to the research examining structural determinants of health among those who experience IPV. The current study found that more restrictive firearm possession policies with relinquishment requirements at the state-level were associated with lower odds of nonfatal injuries. Firearm possession policies may provide upstream opportunities to reduce IPV injuries by potentially disrupting the mechanism of injury. Policies that prohibit firearms from potentially high-risk groups of perpetrators may help reduce adverse injury outcomes among victims.

## Data Availability

Some of the data that support the findings of this study are available from Inter-university Consortium for Political and Social Research, but restrictions apply to the availability of these data, which were used under license for the current study, and so are not publicly available. Data are however available from the authors upon reasonable request and with permission of Inter-university Consortium for Political and Social Research.
